# Rice Hull Extract (RHE) Suppresses Adiposity in High-Fat Diet-Induced Obese Mice and Inhibits Differentiation of 3T3-L1 Preadipocytes

**DOI:** 10.3390/nu11051162

**Published:** 2019-05-24

**Authors:** Ga-Hee Kim, Jae-Yun Ju, Kyung-Sook Chung, Se-Yun Cheon, Tae-Young Gil, Divina C. Cominguez, Yun-Yeop Cha, Jong-Hyun Lee, Seong-Soo Roh, Hyo-Jin An

**Affiliations:** 1 Department of Pharmacology, College of Korean Medicine, Sangji University, Wonju-si 220702, Korea; Chirs35@naver.com (G.-H.K.); soakma@daum.net (J.-Y.J.); adella76@daum.net (K.-S.C.); chunsay1008@naver.com (S.-Y.C.); sophia14t@gmail.com (T.-Y.G.); divina_0406@yahoo.com (D.C.C.); 2 Department of Rehabilitation Medicine of Korean Medicine and Neuropsychiatry, College of Korean Medicine, Sangji University, Wonju-si 220702, Korea; omdcha@sangji.ac.kr; 3 Department of Pharmacy, College of Pharmacy, Dongduk Women’s University, Seoul 03084, Korea; naturalmed@dongduk.ac.kr; 4 Department of Herbology, Daegu Haany University, Daegu 42158, Korea; ddede@dhu.ac.kr

**Keywords:** rice hull extract, high-fat diet, MAPK, obesity, AMP-activated protein kinase

## Abstract

Obesity is one of major health challenges in the industrial world. Although rice hull has been reported to show various bioactivities, no studies have evaluated its anti-obesity effect. We hope to demonstrate the anti-obesity effect of rice hull extract (RHE) and the underlying mechanism in high-fat diet (HFD)-induced obese mice and 3T3-L1 preadipocytes. Serum lipid profiles were determined by enzymatic methods. Histological analysis of liver and epididymis fat tissues was carried out with hematoxylin and eosin stain. The mRNA expression of adipogenic markers was analyzed with qRT-PCR and western blotting. Oral administration of RHE reduced body weight gain and fat accumulation in HFD-fed mice. RHE also reduced lipid accumulation by inhibiting the mRNA expression of adipogenic-related genes in HFD-fed obese mice and differentiated preadipocytes. The downregulation of adipogenesis by RHE was mediated through the phosphorylation of AMP-activated protein kinase (AMPK) and acetyl-CoA carboxylase (ACC). In addition, RHE induced the phosphorylation of c-Jun N-terminal kinases (JNK) and extracellular-signal-regulated kinases (ERK) in liver and epididymis adipose tissues of HFD-fed obese mice. Taken together, these findings indicate that RHE could inhibit the differentiation of adipose cell and prevent HFD-induced obesity, suggesting its potential in the prevention of obesity and metabolic syndrome and related-disorders.

## 1. Introduction

Obesity is a multifactorial chronic disease that is associated with a number of risks. This disease is a risk factor for the development of different conditions such as Diabetes mellitus type 2, coronary thrombosis, cancer, and osteoarthritis [1]. Progression of obesity is characterized by an increase in both the number and mass of fat cells in fat [1]. Adipocytes deposit surplus energy in the form of triglycerides and release it as fatty acid and glycerol. For that reason, changed adipocyte use has influences on metabolic diseases [2]. Uncontrolled increase in the size and number of adipocytes leads to lipid deposition. Adipocytes’ main role is regulating energy homeostasis in conditions like metabolic syndrome and obesity [3]. A complicated interaction between the proliferation and differentiation of preadipocytes influences the rate of adipogenesis [4]. Therefore, regulation of adipogenesis with the use of natural products is an option that can be explored in the prevention of obesity.

Multiple transcription factors are concerned in the differentiation of adipocytes. These factors regulate the expression of various genes that are responsible for the development of mature adipocytes [5]. There are three known major transcription factors of adipogenesis: nuclear receptor peroxisome proliferator-activated receptor gamma (PPARγ), CCAAT/enhancer binding protein-alpha (C/EBPα), and sterol regulatory element-binding transcription factor 1 (SREBP1). They are crucial in the intricate transcriptional cascade of adipocyte differentiation [6]. Insulin, glucocorticoids, and cAMP-elevating agents are regarded as adipogenic stimulators. These stimulators induce the expression of some transcription factors, subsequently upregulating PPARγ. The upregulated expression of PPARγ induced the C/EBPα and subsequently the orchestration of terminal adipogenesis.

A major function of AMP-activated protein kinase (AMPK) is energy homeostasis, and its activation is involved in the regulation of differentiation [7]. ACC, which is one of the target molecules of AMPK, is a necessary enzyme for fatty acids homeostasis [8]. Generally, it is believed that AMPK can turn over from anabolic pathways to catabolic pathways by inhibiting the expressions of genes related to adipogenesis, such as PPARγ [9,10]. Intracellular mitogen-activated protein kinase (MAPK) signaling pathways play an important role in the regulation of cell proliferation and differentiation [11]. It is possible that the MAPK pathway is an available target for the treatment of obesity based on the mechanism by which preadipocytes differentiate into adipocytes [12]. 

Natural products are potential sources of therapeutic agents for obesity and related diseases [13]. Rice (*Oryza sativa* L.) is the main source of food in many countries besides the Asia. Half of the world’s population depends on rice as a staple, making it the second-leading global cereal crop [14]. Rice hull is a byproduct of rice alongside rice bran and they account for 20% of agricultural byproducts. However, it is necessary to protect rice seeds during growth [15]. Additionally, rice hull consists mostly of lignin, hemicellulose, cellulose, and hydrated silica [16]. 

In Cambodia, rice hull (husk) has been used for treating dysentery [17]. Besides, it has been reported to have various pharmacological effects, including antioxidant, anti-inflammatory, and anti-allergic properties [16,18]. Furthermore, water extract of rice hull (RHE) protects insulin-producing islet cells from damage via inflammation and oxidative stress in high-fat diet (HFD)-fed mice [19]. In addition, we found out that RHE regulates the disparity between prostatic cell growth and apoptosis in a rat model of testosterone-induced benign prostatic hyperplasia [20]. Although rice hull has diverse bioactivities, the molecular basis for its antiobesity effects is poorly described. In the present study, HFD-induced obese animals and differentiated 3T3-L1 preadipocytes were used as models of adipogenesis and obesity to investigate the molecular mechanism of the anti-adipogenic properties of RHE.

## 2. Materials and Methods

### 2.1. Chemicals and Reagents

Isobutyl methyl xanthine (IBMX), dexamethasone (DEX), insulin, Oil red O, and all other chemicals were purchased from Sigma-Aldrich (St. Louis, MO, USA). Dulbecco’s modified eagle’s medium (DMEM), bovine serum (BS), heat-inactivated fetal bovine serum (FBS), and penicillin–streptomycin (PS) were purchased from Life Technologies, Inc. (Grand Island, NY, USA). Antibodies for phospho-adenosine monophosphate-activated protein kinase α (p-AMPKα), AMPK, phospho-acetyl-CoA carboxylase (p-ACC), ACC, extracellular signal-regulated kinases (ERK), c-Jun N-terminal kinases (JNK), PPARγ, C/EBPα, and β-actin were purchased from Santa Cruz Biotechnology, Inc. (Santa Cruz, CA, USA). Horseradish peroxidase-conjugated secondary antibodies were obtained from Jackson Immuno Research laboratories, Inc. (West Grove, PA, USA). SYBR^TM^ Green PCR Master Mix was purchased from Applied Biosystems^TM^ (Foster City, CA, USA). Oligonucleotide primers of PPAR-γ, SREBP1, C/EBPα, and GAPDH were purchased from Bioneer (Daejeon, Chungbuk, Republic of Korea).

### 2.2. Preparation of RHE

Rice, *O. sativa* L., was obtained from Hultea Bio Co. (Wonju, Gangwon, Republic of Korea). We have reported the preparation of RHE in a previous study [20]. In brief, rice hulls (300 g) were weighed and soaked in 3 L of water and left overnight. Then, the rice hull was boiled for 1 h, and the water extract was filtered using Whatman No. 1 filter paper. The filtrate was concentrated under reduced pressure on an EYELAN-1000 rotary evaporator (EYELA Riakikai Co., LTD, Tokyo, Japan) at 40 °C. Finally, 30 g of dry extract was obtained from 300 g of rice hull (10 % yield) and stored at −20 °C for later use.

### 2.3. Animal Experiments

Twenty-four four-week-old C57BL/6J mice (male, 23.22 ± 1.67 g) were purchased from Daehan Biolink Co. (Daejeon, South Korea). The mice had adequate access to food and water while being transported. Thereafter, they were maintained (six mice per cage) in a 12-h light/dark cycle at 22 ± 3 °C and a relative humidity of 45 ± 5% in accordance with the guidelines of National Institutes of Health. The experiment was approved by the ethical committee on animal care and the use of laboratory animals in Sangji University (Registration no. 2014-14). We made an effort to minimize the suffering of mice in all the experimental methods of the animal study. Mice were given free access to food and water for one week. After one week of acclimatization, mice were randomly divided into four groups (*n* = 6 per group) by an extraneous researcher: the control group (CON), HFD (45 kcal% fat diet, D12451, Table S1), HFD supplemented with 20 mg/kg of RHE (RHE 20), and HFD supplemented with 40 mg/kg of RHE (RHE 40). The mice in the CON group and the HFD group were fed a commercial standard chow diet and HFD, respectively, for 14 weeks. RHE was dissolved in PBS and was orally and daily administered for 14 weeks along with HFD in the RHE 20 and RHE 40 groups. Body weights were measured and recorded every week. Prior to experimental analysis, the mice were fasted overnight. The following day, animals were anesthetized with Zoletil 50 (20 mg/kg i.p.) according to the manufacturer’s instruction (Virbac, Carros Cedex, France); serum was collected, and lipid levels were measured. The mice were then sacrificed; liver and epididymis fat tissues were excised immediately and stored at −80 °C.

### 2.4. Serum Analysis

During blood sample collection, animals were already in terminal anesthesia. Blood samples were collected by cardiac puncture. The samples were centrifuged at 1005× *g* for 10 min to obtain serum samples. The concentrations of triglyceride (TG) and those of the two main types of cholesterol—low-density lipoproteins (LDL)-cholesterol and high-density lipoprotein (HDL)— were measured by enzymatic methods with a mercantile available assay kit (BioVision Research Products, Inc., Milpitas, CA, USA).

### 2.5. Histological Analysis

Liver and epididymis fat tissues were fixed with 3.7% formaldehyde solution (10% formalin) and embedded in paraffin. For histological analysis, sections of the samples (8 μm thickness) were stained with hematoxylin and eosin (H&E). The stained slides were observed with a Leica DM IL LED microscope (Leica, Wetzlar, Germany).

### 2.6. Western Blot Analysis

Tissues and 3T3-L1 cells were homogenized with the PRO-PREP^TM^ protein extraction solution (Intron Biotechnology Inc., Seongnam, Republic of Korea) and incubated for 10 min on ice to induce cell lysis. The lysates were centrifuged at 11,000× *g* for 20 min at 4 °C, and the supernatant was transferred to a 1.5-mL microtube. The concentrations of protein in the samples were measured using the Bio-Rad protein assay reagent according to the manufacturer’s instruction (Bio-Rad, California, CA, USA). Aliquots of each sample (10–30 µg protein) were separated on an 8%–10% SDS-polyacrylamide gel and then transferred to a polyvinylidene difluoride (PVDF) membrane. The membrane was incubated for 1 h with 2.5% or 5% skim milk at 22 ± 3 °C, followed by overnight incubation with primary antibodies (1:1000 dilutions) at 4 °C. The membranes were washed thrice with Tween 20/Tris-buffered saline (T/TBS). Next, they were incubated with horseradish peroxidase-conjugated secondary antibodies (1:2500 dilutions) for 2 h at 22 ± 3 °C. After rinsing the blots three times with T/TBS, the immuno-detection bands were developed with ECL solution (Ab Signal, Seoul, Republic of Korea).

### 2.7. Real-Time PCR Analysis

After homogenization of the cells and tissues, total RNA was isolated with Easy-Blue Reagent (Intron Biotechnology Inc, Seongnam, Republic of Korea) according to the manufacturer’s instructions. Quantification of total RNA was conducted with an Epoch microvolume spectrophotometer system (BioTek Instruments Inc., Winooski, VT, USA). cDNA was obtained using isolated total RNA (1 μg), d(T)16 primer, and AMV reverse transcriptase. Relative gene expression was quantified using real-time PCR technique (Real Time PCR System 7500, Applied Biosystems) with SYBR Green PCR Master Mix (Applied Biosystems) and oligonucleotide primers (Table 1) purchased from Bioneer. The threshold cycle (Ct) values of PPARγ, C/EBPα, and SREBP1 genes were normalized to the Ct values of GAPDH using gene express 2.0 program (Applied Biosystems). We used the comparative Ct method to calculate the fold changes in gene expression. 

### 2.8. Cell Culture and Adipocyte Differentiation

3T3-L1 mouse preadipocytes were cultured in DMEM supplemented with 10% BS, 100 units/mL penicillin, and 100 μg/mL streptomycin at 37 °C in 5% CO_2_. Two days after the cells had reached confluence (d0), they were stimulated with methylisobutylxanthine, dexamethasone (DEX), and insulin (MDI) induction medium (DMEM containing 10% heat-inactivated FBS, 0.5 mM IBMX, 1 μm/mL DEX, and 1 μg/mL insulin) and were treated with various concentrations of RHE (25, 50, and 100 μg/mL). Three days after stimulation with MDI (d3), the cells were cultured in an insulin medium (DMEM containing 10% heat-inactivated FBS and 1 μg/mL insulin) and were treated with various concentrations of RHE. Three days later (d6), the cells were cultured in 10% FBS/DMEM and were treated with various concentrations of RHE. Full differentiation was achieved by d8. 

### 2.9. Oil Red O Staining

The differentiated cells were fixed with 10% formalin for 60 min. Thereafter, the fixing solution was discarded and the cells were incubated in a staining solution of Oil red O (Sigma Chemical Co., St. Louis, MO, USA) for 3 h. Next, the cells were destained with 40% isopropanol. Oil red O was eluted by incubating the cells in 1 mL of 100% isopropanol for 10 min. Lipid accumulation in the samples was quantified by measuring the optical densities of the samples in a spectrophotometer at 520 nm.

### 2.10. Statistical Analysis

Data are expressed as mean ± standard deviation (SD) of three experiments. All analyses were performed with Prism 5 (GraphPad Software Inc. v.5.01, San Diego, CA, USA). Statistical significance was determined with ANOVA and Dunnett’s post hoc test. Differences between the groups were considered to be significant at *p* < 0.05.

## 3. Results

### 3.1. RHE Reduced Body Weight Gain and Suppressed Lipid Droplet Accumulation in Liver and Epididymis Adipose Tissue

**Figure 1:**
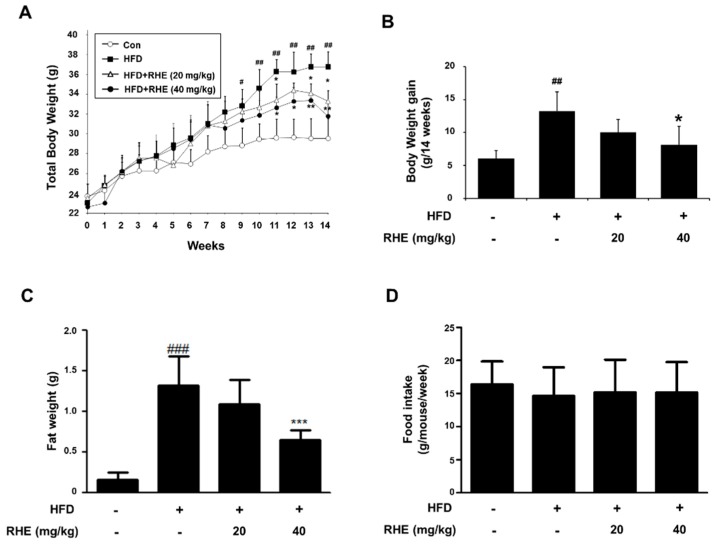
Effect of rice hull extract (RHE) on body weight and serum lipid profile in high-fat diet (HFD)-fed mice. Mice were fed HFD for 14 weeks in the presence or absence of RHE (20 or 40 mg/kg/day). Changes in (**A**) total body weight, (**B**) body weight gain, (**C**) fat pad weight of epididymal adipose tissue, (**D**) food intake in each group are shown. The data shown mean ± S.D. ^#^ *p* < 0.05, ^##^ *p* < 0.01 and ^###^ *p* < 0.001 vs CON group; * *p* < 0.05, ** *p* < 0.01 and *** *p* < 0.001 vs HFD group.

No toxicity, mortality, or harmful effects were observed. We compared the body weight, fat weight, and lipid profile with each group. As shown in Figure 1A, the total body weight of mice in the HFD group was higher than that of mice in the CON group. In addition, body weight gain of the HFD group was higher than that of the CON group (54.39%, *p* < 0.01, Figure 1B). In contrast to HFD feeding, RHE administration suppressed body weight gain by 24.2% and 37.9% (*p* < 0.05) in the RHE 20 and RHE 40 groups, respectively. RHE treatment also reduced fat weight in the HFD group (Figure 1C). The serum TG, LDL-cholesterol, and HDL-cholesterol were also analyzed and are presented in Table 2. In comparison with CON group, HFD group had significantly increased TG and LDL-cholesterol. In the RHE 40 group, however, the serum TG and LDL-cholesterol significantly decreased, whereas the serum level of HDL-cholesterol was not affected by RHE treatment. 

### 3.2. RHE Ameliorated Adiposity and Fat Accumulation in HFD-Fed Obese Mice

**Figure 2:**
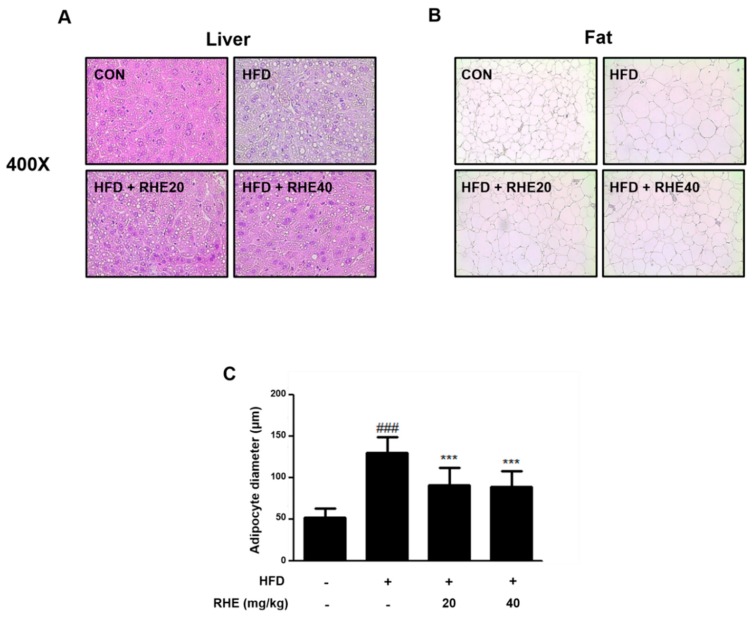
Effect of RHE on fat accumulation in HFD-fed mice. Representative histological images of (**A**) the liver and (**B**) epididymal adipose tissue were assessed using H&E staining (original magnification; (A) 400× and (B) 200×, respectively). (**C**) Adipocyte diameter was quantified under microscope quantified from representative sections. The data shown represent mean ± S.D. of three independent experiments. ^###^ *p* < 0.001 vs CON group; *** *p* < 0.001 vs HFD group.

To measure the effect of RHE on the histological change, we stained tissue samples with H&E. As shown in Figure 2A, while liver sections of the HFD group exhibited lipid deposition in the hepatocytes, particularly around the central vein, there was a reduced deposition of lipids in the RHE-treated liver samples. In addition, the analysis of adipose tissue accumulation in epididymis adipose tissue revealed that RHE ameliorated the accumulation of lipids and reduced the size of adipocytes in HFD-fed obese mice (Figure 2B,C, *p* < 0.001). These data suggested that RHE was effective in preventing HFD-induced obesity via its effects on body weight, epididymis fat mass, and fat accumulation. 

### 3.3. RHE Suppressed mRNA Expression of Adipogenic Genes in HFD-Fed Obese Mice

**Figure 3:**
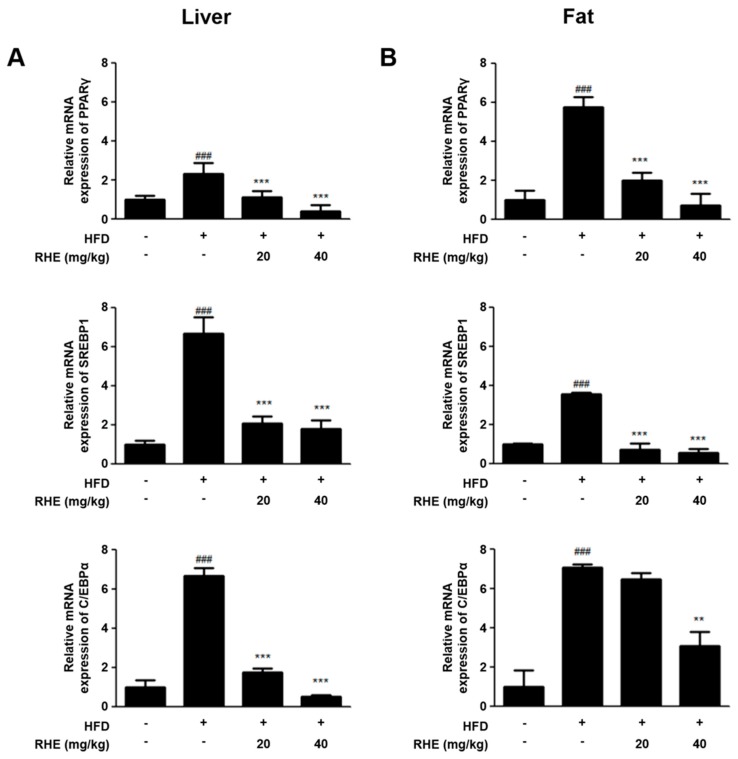
Inhibitory effects of RHE on the mRNA expression level of adipogenesis-related genes in (**A**) liver and (**B**) adipose tissue of HFD-fed obesity mice. The mRNA expression of PPARγ, SREBP1, and C/EBPα was analyzed by quantitative real-time PCR. Data were modified to the GAPDH mRNA levels and compared to CON group’s measurements assigning a value of 1.0. The data shown mean ± S.D. of three independent experiments. ^###^ *p* < 0.001 vs CON group; ** *p* < 0.01 and *** *p* < 0.001 vs HFD group.

PPARγ, SREBP1, and C/EBPα are the crucial factors of adipogenesis. These factors are essential for regulating the genes of adipogenesis [21]. To investigate the effects of RHE on the mRNA expression of adipogenic genes induced by HFD in liver tissue and epididymis adipose tissue, we conducted qRT-PCR analysis. HFD-induced mice indicated upregulated mRNA levels of adipogenesis-related genes such as PPARγ, SREBP1, and C/EBPα compared with CON (*p* < 0.001), but these factors were downregulated by RHE in both liver and epididymis adipose tissues (Figure 3).

### 3.4. RHE Activated AMPK in HFD-Fed Obese Mice

**Figure 4:**
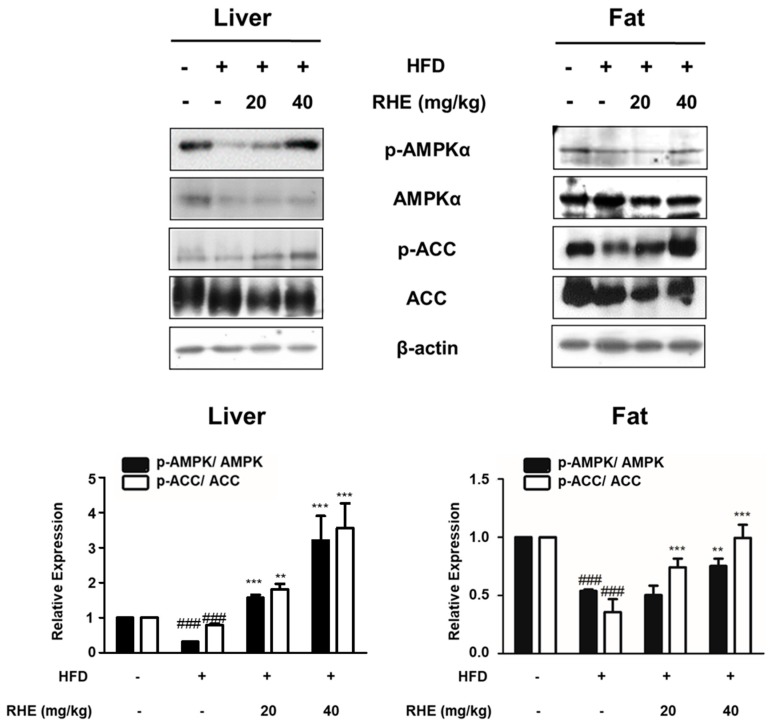
Effects of RHE on phosphorylation of the AMPKα signaling pathway in liver and adipose tissue of HFD-fed obesity mice. The phosphorylation and expression of AMPKα and ACC proteins were detected by western blot analysis with specific antibodies. β-actin was used as internal control. Densitometric analysis was done with Bio-Rad Quantity One^®^ Software. The data represent mean ± S.D. of three independent experiments. ^###^ *p* < 0.001 vs CON group; ** *p* < 0.01 and *** *p* < 0.001 vs HFD group.

A major function of AMPK is energy homeostasis, and its activation is involved in the regulation of differentiation [7]. ACC, which is one of the target molecules of AMPK, is a necessary enzyme for fatty acids homeostasis [8]. Therefore, we investigated if RHE would regulate adipocyte differentiation and energy metabolism via the regulation of AMPK in liver tissues and epididymis adipose tissues of HFD-fed mice. As shown in Figure 4, western blot analysis revealed that the HFD group showed less phosphorylation of AMPK phosphorylation than the CON group (*p* < 0.001), but this level was significantly enhanced by administration of RHE (40 mg/kg) in both liver and epididymis adipose tissues (*p* < 0.001 and *p* < 0.01). Similarly, the phosphorylation level of ACC, a direct downstream target of AMPK, was significantly reduced by HFD, but was reversed in the HFD+RHE samples. These findings revealed that RHE attenuated obesity though activation of the AMPK pathway. 

### 3.5. Effects of RHE on the MAPK Pathway in HFD-Fed Obese Mice

**Figure 5:**
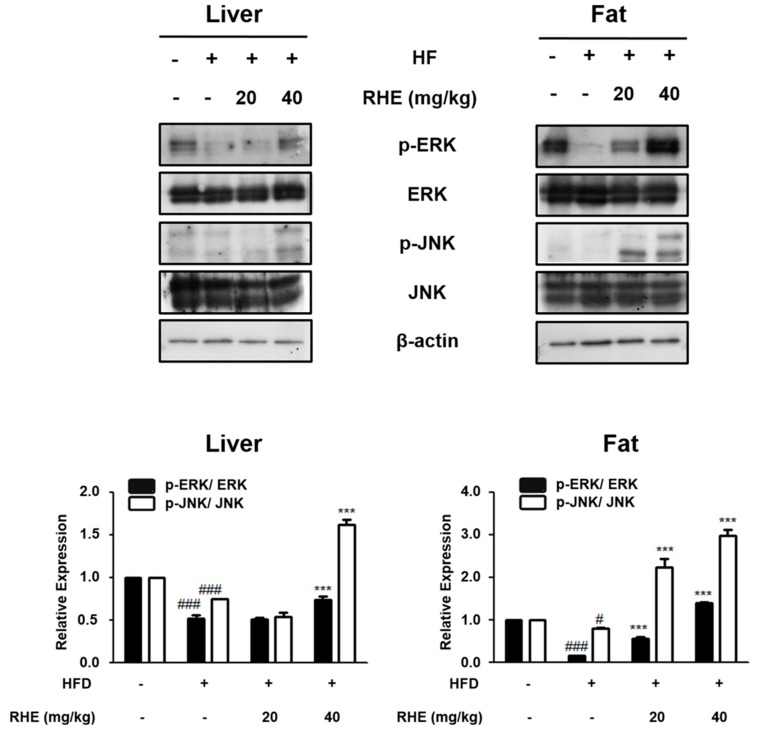
Effects of RHE on activation of the MAPK signaling pathway in liver and adipose tissue of HFD-fed obesity mice. The phosphorylation and expression of ERK and JNK proteins were shown by western blot analysis with specific antibodies. β-actin was used as internal control. Densitometric analysis was performed with Bio-Rad Quantity One^®^ Software. The data were shown mean ± S.D. of three independent experiments. ^#^ *p* < 0.05 and ^###^ *p* < 0.001 vs CON group; *** *p* < 0.001 vs HFD group.

The MAPK pathway is an intracellular signaling pathway that plays a central role in cellular structures and functions, such as differentiation of adipocytes [22]. To evaluate the role of this pathway in the antiobesity activities of RHE, the phosphorylation level of MAPKs was determined by western blot analysis. RHE increased the phosphorylation of ERK and JNK in a dose-dependent manner in both liver and epididymis adipose tissue (Figure 5, *p* < 0.001). However, RHE did not influence the phosphorylation level of p38 (data not shown).

### 3.6. RHE Inhibited Adipogenesis and the Expression of Adipogenic Factors in 3T3-L1 Preadipocytes

**Figure 6:**
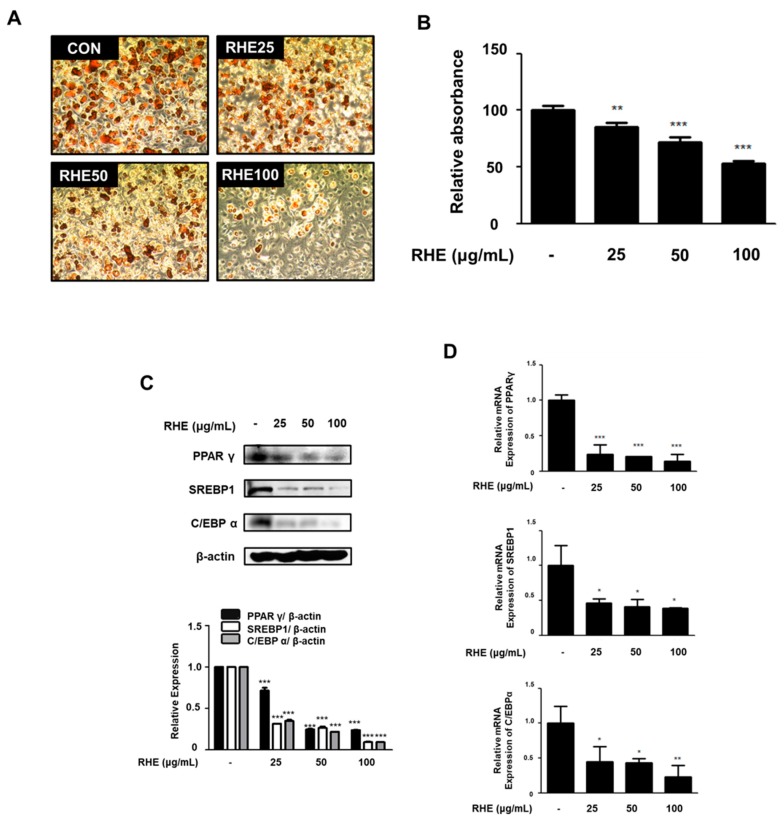
Effects of RHE on lipid accumulation and adipogenic factors in 3T3-L1 preadipocytes. (**A**) 3T3-L1 preadipocytes were treat with RHE at 25, 50, and 100 μg/mL during MDI-induced adipocyte differentiation. The intracellular lipid accumulation was determined with Oil red O staining on Day 8 and the stained cells were photographed at magnification 100×. (**B**) Lipid content was extracted from Oil red O stained cells by isopropanol and quantified spectrometric analysis at 520 nm. (**C**) Protein expression of PPARγ, SREBP1, and C/EBPα was determined by western blot analysis with specific antibodies on Day 8. β-actin was used as internal controls. Densitometric analysis was performed using Bio-Rad Quantity One^®^ Software. (**D**) The mRNA expression of PPARγ, SREBP1, and C/EBPα was analyzed by quantitative real-time PCR on Day 8. Data were standardized to the GAPDH mRNA levels and compared to measurements of differentiated cells. The data shown represent mean ± S.D. of thrice independent experiments. * *p* < 0.05, ** *p* < 0.01, and *** *p* < 0.001 vs. differentiated cells.

To further confirm the effects of RHE on adipogenesis, 3T3-L1 preadipocytes were treated with different concentrations of RHE (25, 50, and 100 μg/mL) during differentiation. RHE treatment caused a significant reduction in Oil red O-stained adipocytes and fat accumulation in differentiated adipocytes compared to the untreated cells (Figure 6A). These results correlated exactly with the absorbance values of eluted Oil red O staining solution in differentiated cells (Figure 6B, *p* < 0.01 or *p* < 0.001). In addition, we analyzed the expression of PPARγ, SREBP1, and C/EBPα using western blot analysis and qRT-PCR analysis in order to investigate whether RHE inhibits the expression of adipogenic factors in MDI-induced 3T3-L1 cells. As shown in Figure 6C,D, the protein and mRNA expression of PPARγ, SREBP1, and C/EBPα was significantly lower than those of the untreated cells.

## 4. Discussion

Our previous study showed that RHE contains many bio-active compounds [20]. GC-MS analysis of RHE showed that 2-methoxyphenol, 4-vinylphenol, 2-methoxy-4-vinylphenol, 4-hydoxybenzaldehyde, and 2,6-dimethoxyphenol were the most abundant compounds in the extract. HPLC-PAD analysis was used to quantify the level of 4-hydroxybenzaldehyde in the extract. Park et al. reported that 4-hydoxybenzaldehyde is able to reduce adiposity and fat accumulation [23]. 

In this study, RHE, which contains 4-hydoxybenzaldehyde, suppressed adipogenesis in liver and adipose tissue through the MAPK pathway in HFD-fed obese mice models. Administration of RHE improved the lipid profile (Figure 1B,C). Fat accumulation is associated with rises in the TG and LDL-cholesterol contents of the serum and thus it is a symptom of dyslipidemia [24]. Our results indicated that RHE supplementation remarkably reduced the levels of serum TG and LDL-cholesterol as the concentration of RHE. These data demonstrated that RHE downregulated the levels of plasma TG and LDL-cholesterol and suppress dyslipidemia in HFD-fed obese models. 

Adipocytes, also known as lipocytes and fat cells, control lipid metabolism using lipogenic factors for differentiation [25]. It means regulation of adipocyte differentiation is crucial for managing obesity at the cellular level. One of the features of adipocyte differentiation is the different stages involved in its process; early, intermediate, and terminal stages. Every stage of an adipogenesis model has an intricate network of key transcription factors such as PPARγ, SREBP1, and C/EBP families [26]. At the early stage of adipocyte differentiation, C/EBPβ and C/EBPδ activate the expression of PPARγ, generating C/EBPα. Upregulation of PPARγ and C/EBPβ alone or in collaboration induces the transcription of many adipogenesis-related genes, leading to the adipocyte phenotype in the late phase [27]. PPARγ belongs to a nuclear receptor superfamily, which also includes PPARα and PPARδ. It forms a heterodimer with other nuclear receptors to regulate the expression of PPAR elements-containing genes associated with lipid or carbohydrate metabolism in adipocytes [28]. C/EBPα regulates insulin sensitivity in adipocytes [29] and plays a pivotal role in SREBP1 expression in the late stage of adipogenesis. Among the identified isomers of SREBP, SREBP1 is the most important lipogenic gene involved in the encoding of enzymes in the synthesis of triglyceride or desaturation of fatty acids [30]. Administration of RHE suppressed the expression of genes that are associated with adipogenesis (PPARγ, SREBP1, and C/EBPα) in HFD-fed obese animal models and 3T3-L1 cells (Figure 3 and Figure 6C,D).

In addition, advancement of the lipid panel in the obese mice model means that RHE has the potential to inhibit lipogenesis. These data suggest that RHE is able to reduce the accumulation of fat by suppressing adipogenesis and lipogenesis. Furthermore, administration of RHE activated AMPKα in liver and epididymis adipose tissues of HFD-fed stout mice after inactivation of ACC (Figure 4). AMPK subsists in various cells as a heterotrimeric complex with a catalytic (α) and two regulatory subunits (β and γ). It is a serine/threonine protein kinase with several isoforms that are made up of different combined subunits and these subunits vary across different tissues [31]. Many studies have demonstrated that activation of AMPK is improved in several diseases [32,33,34]. AMPK is activated by different signaling pathways (e.g., adiponectin, transforming growth factor-β). Moreover, activated AMPK suppresses energy-storage processes via lipogenesis, synthesis of protein, and gluconeogenesis [35]. It is known that the activation of AMPK is enough to hinder adipogenesis by downregulating a few adipocyte-specific transcription factors like PPARγ and C/EBPα [36]. AMPKα deactivates ACC, a fatty acid synthetic enzyme, which lowers malonyl-CoA synthesis and subsequently leads to the activation of CPT-1 (an enzyme involved in β-oxidation) and ultimately diminishes accumulation of TG [37]. This indicates that, in the current study, RHE-activated AMPKα caused the inactivation of ACC and consequently, the oxidation of fatty acid. Thus, RHE was able to decrease lipid accumulation in the epididymis adipose tissues and the liver of HFD-fed obese mice. On the other hand, FAS is one of main lipogenic enzymes in obesity [38]. Since we focused on the relationship between AMPK and ACC, we assumed the AMPK could affect other lipolytic enzymes of upstream signaling pathways.

Intracellular MAPK signaling pathway is a critical factor in controlling cell proliferation and differentiation [22]. In the early stage of adipocyte differentiation, the MAPK pathway is involved in the regulation of the expression of PPARγ and C/EBPα. The pathway influences the expression and phosphorylation of proteins that are associated with adipogenesis [39] and thus is a potential therapeutic target for obesity. ERK is important for the differentiation of preadipocytes; however, the sustained activation of ERK impedes the differentiation process through the activation of PPARγ [22]. Hence, activation of ERK has to be controlled during the differentiation of adipocytes. Similarly, activation of JNK also hinders the differentiation of adipocytes through the phosphorylation of PPARγ and negatively controls its transcriptional activity [40]. Our data showed that RHE enhanced the phosphorylation of ERK and JNK (Figure 5), whereas both had no effect on p38 activation in liver tissue and epididymis adipose tissue of HFD-fed obese mice (data not shown). These findings reveal that the regulatory effects of RHE on the differentiated adipocytes, and its ability to enhance the phosphorylation of ERK and JNK resulted in the downregulation of PPARγ. However, our study had limitations. We focused on the anti-adipogenic effects of RHE on only the adipogenic system via the AMPK and MAPK pathways. However, AMPK signaling pathways are involved in various physiological and metabolic systems, not only the adipogenic system [3]. There are many studies showing that AMPK has a critical role in reprogramming energy metabolism by inhibiting glycolysis via its action on mTOR and p53 [41]. In the previous study, JY Yang, et al. showed the effect of RHSE on glucose-regulating mechanisms [1]. Following this study, we are able to investigate the effect of RHE on glucose metabolism via various signaling pathways besides AMPK.

## 5. Conclusions

RHE demonstrated an antiobesity effect in an HFD-induced obesity mouse model through the regulation of adipogenesis and lipogenesis. Therefore, RHE is a potential agent for the prevention of obesity.

## Figures and Tables

**Table 1:** Primer sequences and PCR conditions.

Genes	Tm (°C)	Size (bp)	Sequence 5′-3′
*PPAR* *γ*	55	148	F: TTCGGAATCAGCTCTGTGGA
	55		R: CCATTGGGTCAGCTCTTGTG
*SREBP1c*	55	115	F: ATCGCAAACAAGCTGACCTG
	55		R: AGATCCAGGTTTGAGGTGGG
*C/EBPα*	55	187	F: TCGGTGCGTCTAAGATGAGG
	55		R: TCAAGGCACATTTTTGCTCC

**Table 2:** Serum biochemical parameters.

Indexes	ND	HFD	HT	
			20 mg/kg	40 mg/kg
Triglyceride (mg/dL)	55.00 ± 10.00	71.67 ± 11.55 ##	58.13 ± 9.98	45.00 ± 8.86
LDL-cholesterol (mg/dL)	2.25 ± 0.87	11.00 ± 1.22 ###	9.67 ± 2.50	9.00 ± 1.15 *
HDL-cholesterol (mg/dL)	67.50 ± 2.89	72.50 ± 2.89	75.50 ± 7.07	78.75 ± 4.79

The data shown mean ± S.D. of thrice independent experiments. ## *p* < 0.01 and ### *p* < 0.001 vs CON group; * *p* < 0.05 vs HFD group.

## Supplementary Materials

The following are available online at https://www.mdpi.com/2072-6643/11/5/1162/s1, Table S1: Ingredient composition of each of diet.

## Abbreviations

RHE: Rice Hull Extract; HFD: High-Fat Diet; AMPK: Adenosine Monophosphate-activated Protein Kinase; PPARγ: Peroxisome Proliferator-Activated Receptor gamma; C/EBPα: CCAAT/enhancer Binding Protein-alpha; SREBP1: Sterol Regulatory Element-Binding transcription factor 1; ACC: Acetyl-CoA Carboxylase; ERK: Extracellular signal-regulated kinases; JNK: c-Jun N-terminal kinases; TG: Triglyceride; LDL: Low-Density Lipoproteins; HDL: High-density lipoproteins; MDI: Methylisobutylxanthine, DEX, and insulin.
