# Supplementary material for: Rice Hull Extract (RHE) Suppresses Adiposity in High-Fat Diet-Induced Obese Mice and Inhibits Differentiation of 3T3-L1 Preadipocytes
Source: Nutrients. 2019 May 24;11(5):1162. doi: 10.3390/nu11051162 (PMC6566172; doi:10.3390/nu11051162)
Supplement: Supplementary file 1 [file nutrients-11-01162-s001.pdf]

nutrients

Supplementary Table S1. Ingredient composition of each diet.

Content	Normal diet	Content	HFD
	% kcal		% kcal
Protein	20.8	Protein	20
Carbogydrate	57.7	Carbogydrate	35
Fat	11.5	Fat	45
**Ingredient**	gm	**Ingredient**	gm
Casein	200	Casein	200
L-cystine	0	L-cystine	3
Corn starch	150	Corn starch	72.8
Maltodextrin10	0	Lodex 10	100
Sucrose	500	Sucrose	176.8
Cellulose, BW200	50	Solka floc, FCC200	50
Corn oil	50	Soybean oil	50
Lard	0	Lard	177.5
Mineral Mix S10001	35	Mineral Mix S10026B	50
Vitamin Mix V10001	10	Vitamin Mix V10001	1g
Choline Bitartrate	2	Choline Bitartrate	2
		FD&C Red Dye #40	0.05
